# Emotional Intelligence Profiles and Mobbing in Nursing: The Mediating Role of Social Support and Sensitivity to Anxiety

**DOI:** 10.3390/ejihpe11020026

**Published:** 2021-04-06

**Authors:** María del Mar Molero Jurado, África Martos Martínez, Ana Belén Barragán Martín, María del Mar Simón Márquez, Nieves Fátima Oropesa Ruiz, Maria Sisto, María del Carmen Pérez-Fuentes, José Jesús Gázquez Linares

**Affiliations:** 1 Department of Psychology, Faculty of Psychology, University of Almería, 04120 Almería, Spain; mmj130@ual.es (M.d.M.M.J.); amm521@ual.es (Á.M.M.); abm410@ual.es (A.B.B.M.); msm112@ual.es (M.d.M.S.M.); foropesa@ual.es (N.F.O.R.); ms168@ual.es (M.S.); mpf421@ual.es (M.d.C.P.-F.); 2 Department of Psychology, Universidad Autónoma de Chile, Providencia 7500000, Chile

**Keywords:** social support, nursing, emotional intelligence, mobbing, sensitivity to anxiety

## Abstract

The prevalence of mobbing among nurses in various countries is around 17–20%. Some researchers have attempted to explain the success or failure of adaptation to the work environment and teamwork and to buffer the effects of psychological harassment in the workplace by incorporating emotional intelligence into the mobbing context. As its main objectives, this quantitative, observational, cross-sectional study analyzed the relationship between emotional intelligence and mobbing as perceived by nurses and sought to establish the mediating roles of other variables involved, such as social support and sensitivity to anxiety. The final sample consisted of 1357 Spanish, self-selected nurses aged 22–58 from multiple healthcare institutions. The questionnaires (Perceived Psychological Harassment Questionnaire, The Brief Emotional Intelligence Inventory, Brief Perceived Social Support Questionnaire, Anxiety Sensitivity Index-3) were implemented on a web platform, which enabled the participants to complete them online. Descriptive analyses and mediation models were estimated. Personal characteristics related to high sensitivity to anxiety and low emotional intelligence implied greater presence of mobbing at work. This mobbing may be buffered if the person perceives enough support from family, friends or significant others. Our results recommend reinforcing the social support network of nursing personnel to improve the work climate and training them in emotional intelligence in university and on-the-job programs.

## 1. Introduction

Mobbing refers to the phenomenon by which one or several persons in the workplace exert extreme psychological harassment toward another or others by destroying their communication networks and their professional reputation and impairing their physical or psychological health so that they quit their jobs [1]. Thus, mobbing refers to a type of bullying or passive harassment in the workplace by an individual or an entire team, which causes the most profound isolation in the victims, who usually feel unable to demonstrate the attacks and articulate the problem and, if they do so, may lack the support of their coworkers [2]. Although the term bullying is more common in research, there is a need to understand and approach mobbing as a separate concept from abuse by classmates, as its development, socioeconomic consequences and the imbalance of power differ [3]. In this sense, the negative consequences of mobbing for healthcare workers, the healthcare system and the quality of patient care have been widely demonstrated [4,5,6,7], although cultural, economic, legal, organizational and other factors should also be considered for their prevention and coping [8]. Such maladjustment in relations with others, if continued, generally leads to a distortion of how one feels, becoming harmful to one’s health over time.

In Spain, the latest Cisneros report stated that over 34% of nursing personnel had experienced psychological mobbing in the workplace [9]. However, this varies widely depending on whether they are direct or indirect victims of harassment. Thus, its prevalence in nursing personnel during the last decade, considering studies in other countries, would be closer to 17–20% [10].

In view of the above, a change in how a representative percentage of these healthcare professionals relate to each other would be indispensable. It is also urgent to improve interpersonal relations and provide a common space in the workplace for them to meet to contribute to the positive development of the organization and its members, more so because their work deals basically with improving the health of others, for which good psychological adjustment is an indispensable requirement [6,11,12,13].

Mobbing may be attributed to a variety of causes. The most accepted explanatory models of mobbing at work by Leymann [1], Hirigoyen [14] & Piñuel [15] differentiate situational (acting in secret, scapegoating, frightened witnesses), organizational (how work is organized and characteristics of organizational behavior) and abuser traits (paranoid, psychopathic and narcissistic personality characteristics) [16,17]. Their intersection at different stages usually culminates in harming a victim, who ends up quitting. This is not surprising in situations of psychological abuse, where the person is increasingly overcome by fear [18].

Emotional intelligence may be defined as a set of personal and interpersonal emotional skills which influence one’s capacity to cope with environmental demands and pressures [19]. The value of emotional intelligence in predicting psychosomatic and mental health has been corroborated by various authors [11,20]. Some researchers [21,22] explain success or failure in adapting to the workplace or teamwork by incorporating psychological variables, such as emotional intelligence, in the mobbing context to buffer the effects of mobbing. They also emphasize the dynamic, complicated and cyclical nature of workplace mobbing, and the role of affect [22,23,24,25]. Emotional intelligence has a role in education, training and the development of leadership in the organizational context [26], where it is a variable which has been related positively to job satisfaction and commitment [27,28,29]. In view of the above, this study approached mobbing in nursing from a perspective considering the role of emotional intelligence in psychological harassment and its relationship with other psychological, personal and social variables. In this respect, high emotional intelligence has been related to more positive affect and emotions, while lower emotional intelligence could lead to attitudes of isolation at work, since, under such circumstances, it is harder to manage behavior positively [30]. In more detailed studies of emotional intelligence, it has been found to have a moderating role in coping with stress and anxiety [31,32,33]. In anxiety disorders, some components of emotional intelligence act as mediators between sensitivity to anxiety and the manifestation of anxiety symptoms [34]. Applied to the work environment, improved emotional competence has been proposed to reduce work stress and workplace mobbing [35]. Findings suggesting that mindfulness improves emotional intelligence and sensitivity to anxiety are few but hopeful [36,37,38].

Similarly, according to Leymann [1], and in agreement with other authors [39,40,41], a psychological view of harassment is based on a person’s subjectivity; that is, the negative acts in mobbing must be considered hostile. From this perspective, sensitivity to anxiety has a relevant role in interpreting certain hostile behavior, which may not mean the same or generate the same anxiety in one individual as another [42,43,44]. This does not mean that aggression related to mobbing as described by various authors is not significant or representative of psychological harassment, but that there may be a certain amount of variability in how different people interpret this behavior, such that sensitivity to anxiety might be a mediating variable in the relationship between emotional intelligence and violence connected to mobbing. Furthermore, some studies have shown that positive social support can counteract the negative effects of accumulated exposure to harassment [45,46,47], buffering affective processes related to mobbing. Social support has also been positively related to emotional intelligence, particularly with an ability for understanding and emotional regulation [48,49], and is considered a protective factor against burnout in nursing personnel [50,51,52,53]. Nevertheless, in the academic context, Extremera et al. [54] found that emotional intelligence was not correlated with perceived support, but, rather, social support exerted a mediating effect on the relationship between emotional intelligence and teacher participation. Therefore, another of our objectives was to analyze the role of the sensitivity to anxiety and perceived social support variables, in relation to certain emotional profiles and mobbing in the workplace.

Thus, keeping in mind our research objectives, the hypotheses of this study were:

**Hypothesis** **1** **(H1):** *Different profiles can be identified based on emotional intelligence component scores, with differences in social support, sensitivity to anxiety and perceived mobbing.*

**Hypothesis** **2** **(H2):** *An emotional profile with scores below the mean is positively related to perceived psychological harassment*.

**Hypothesis** **3** **(H3):** *Perceived social support acts as a mediator (protective effect) in the relationship between the emotional profile and perceived mobbing*.

**Hypothesis** **4** **(H4):** *Sensitivity to anxiety acts as a mediator (risk effect) in the relationship between the emotional profile and perceived mobbing*.

**Hypothesis** **5** **(H5):** *Social support reduces the harmful effect of sensitivity to anxiety on the relationship between the emotional profile and perceived mobbing*.

**Figure 1:**
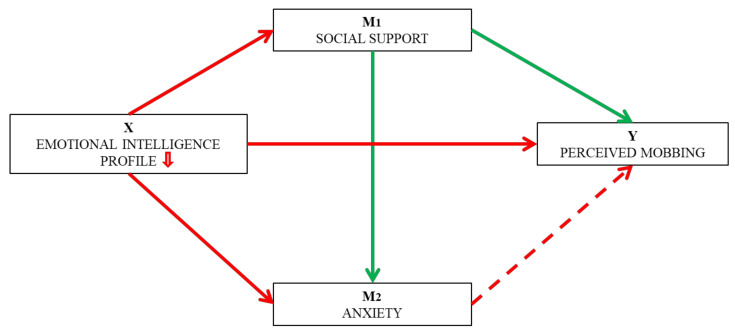
Hypothesized model of the mediation effects of social support and anxiety on the relationship between emotional intelligence profiles and perceived mobbing. Note: Continuous red line represents a risk effect (H2), continuous green line represents a protective effect (H3), and discontinuous red line represents the risk effect mediated by anxiety sensitivity (H4).

Figure 1 shows the hypothesized model of the mediating effects of social support and anxiety on the relationship between emotional intelligence profiles and perceived psychological bullying.

## 2. Materials and Methods

### 2.1. Participants

The original sample consisted of 1377 Spanish, self-selected nurses. Twenty questionnaires were discarded due to incongruent or random answers to the control items included. Thus, the final sample was *n* = 1357 nurses aged 22–58 (M = 30.86, SD = 6.09), the majority being women (83.9%, *n* = 1138). At the time of data collection, 23.1% were working under a permanent contract, 72.2% (*n* = 980) temporary, and the remaining 4.7% (*n* = 64) were unemployed.

### 2.2. Instruments

An ad hoc questionnaire was prepared to collect sample sociodemographic data (age, sex) and information on their current employment status.

The Perceived Psychological Harassment Questionnaire (CAPP) [55] evaluates psychological harassment in the workplace with 15 items rated on a Likert-type scale (from 1 = “I do not feel like that at all” to 5 = “I always feel like that”). It includes items intended to provide a more general evaluation of the subject (e.g., “I feel as if I were being bullied at work”) and others referring to more specific episodes in an environment characterized by perceived psychological harassment (e.g., “Some staff members openly despise and taunt others”). The higher the score on the CAPP, the stronger the feeling of harassment. The reliability coefficient of the CAPP in this study was α = 0.93, which is similar to what the authors found in a sample of professionals from several different areas, healthcare among them [37].

The Brief Emotional Intelligence Inventory (EQ-i-20M) [56] is an adaptation for an adult Spanish population of the Emotional Intelligence Inventory: Young Version (EQ-i-YV) by Bar-On and Parker [57]. It consists of 20 items with four answer choices on a Likert-type scale, providing a score on five emotional intelligence factors: intrapersonal (e.g., “I can describe my feelings easily”), interpersonal (e.g., “I understand how other people feel”), stress management (e.g., “I find it difficult to control my anger”), adaptability (e.g., “I can solve problems different ways”) and mood (e.g., “I feel sure of myself”). The reliability indices were adequate, with a Cronbach’s Alpha of 0.88 for the intrapersonal dimension; 0.77 for interpersonal; 0.77 for stress management; 0.84 for adaptability and 0.90 for mood.

The Brief Perceived Social Support Questionnaire (CASPE) [58] consists of nine items analyzing three affective bonds, from which social structure supports can be deduced by the type of bond. Perceived social support evaluated by the questionnaire is structured on three axes, which could be called: family/significant others (Factor 1); friends/other staff (Factor 2); and, finally, partner/associationism (Factor 3). Factors 1 and 2 are unidimensional and are interpreted directly. Factor 3 is a bipolar construct, with good relations with partner at one end and associationism at the other (this factor was not included in the data analysis due to its mediation characteristics). It therefore evaluates the subject’s perception of support from family (number of contacts and quality of relations), friends and partners or significant others (number of friends, shared activities, perceived functional and emotional support) and provides a total score of 9 to 35. The total score is interpreted such that the higher the score, the stronger social support is. In this case, reliability was α = 0.82.

The Spanish version of the Anxiety Sensitivity Index-3 (ASI-3) [59] by Sandín et al. [60] was used in this study. It has 18 items, which refer to fear/anxiety reactions to physical symptoms, loss of cognitive control and visible symptoms in social situations. The answers are rated on a five-point Likert-like scale (0 = “not at all, or almost not at all” to 4 = “very much”). It provides a general index of sensitivity to anxiety and scores on three subscales: physical (e.g., “It scares me when my heart beats fast”) cognitive (e.g., “It scares me when I am unable to keep my mind on a task”) and social (e.g., “It is important for me not to give the impression that I am nervous”). Reliability for the global scale was 0.94, for the physical anxiety subscale was 0.92, for cognitive anxiety was 0.90 and it was 0.82 for social anxiety.

### 2.3. Procedure

Spanish healthcare centers and scientific societies distributed the survey among their members in their bulletins by email, social networking sites, etc. The questionnaire was implemented on a web platform, which enabled the participants to complete them online during the last quarter of 2019. A series of control questions were included to be able to identify random or incongruent answers, and these cases were discarded from the study sample. The survey was also distributed on social networks (specifically, to healthcare worker profiles by snowball sampling), thereby acquiring access to nurses who were not working at the time of data collection.

### 2.4. Ethical Considerations

Before data were collected, participants were guaranteed compliance with the standards of information, confidentiality and ethics in data processing. Participation was voluntary, and, on the first page, before starting to answer the questionnaire, relevant information on the study and its purpose was provided. The participants gave their informed consent by marking a box designated for the purpose, which then allowed them to continue with the questionnaire. The Bioethics Committee approved the study (Ref: UALBIO2019/031).

### 2.5. Data Analysis

The study design was quantitative, observational and cross-sectional. First, to identify the relationships between the study variables (emotional intelligence, perceived social support and sensitivity to anxiety) and perceived mobbing, the Pearson’s correlation coefficient was calculated in addition to the corresponding descriptive statistics.

A two-stage cluster analysis was performed to identify the profiles by emotional intelligence factor scores. A comparison of means of the groups or clusters identified was then carried out with the Student’s *t* test for independent samples, with a significance level of 0.05, and the Cohen’s *d* [61] to find the effect size of the differences.

Finally, a multiple mediation model was computed to analyze the relationships between the emotional intelligence profile and perceived mobbing, including perceived social support and the general sensitivity to anxiety index as potential mediators. The SPSS macro was used to compute the mediation models [62] with bootstrapping, using 5000 bootstrap samples.

## 3. Results

### 3.1. Correlations and Descriptive Statistics

As observed in the correlation matrix (Table 1), perceived mobbing was positively associated with the total anxiety index and with each of its types (physical, cognitive and social). Similarly, all of the emotional intelligence components (intrapersonal, interpersonal, stress management, adaptability and mood) and perceived social support were also associated significantly but negatively with perceived mobbing.

The relationships between emotional intelligence and anxiety were negative in all cases. The intrapersonal and interpersonal factor associations were significantly different. Specifically, the intrapersonal factor correlated negatively with total anxiety and its social component, while the interpersonal factor correlated negatively with the physical and cognitive components of anxiety. Positive correlations were found between perceived social support and all the emotional intelligence factors, and negatively with respect to sensitivity to anxiety, both with the global index and each of its components.

### 3.2. Emotional Intelligence and Perceived Mobbing: Profiles and Differences

**Figure 2:**
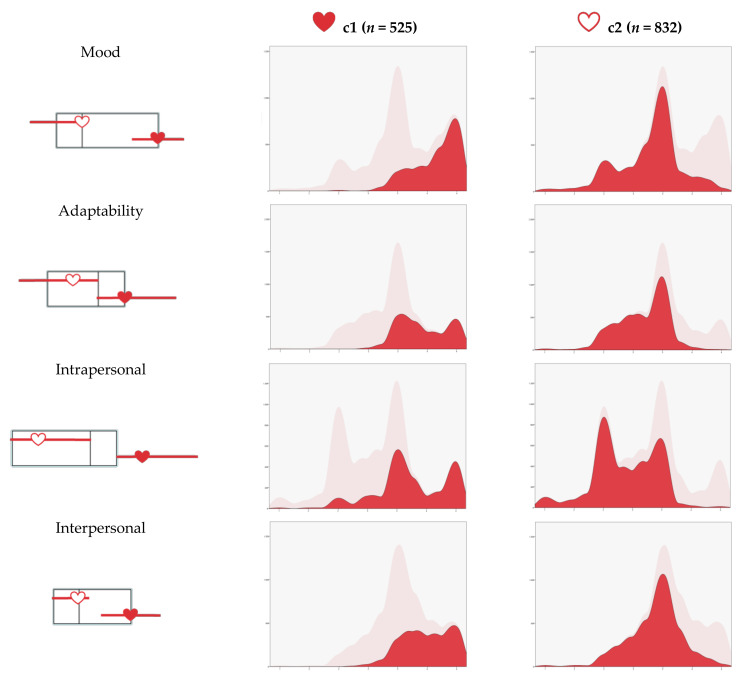

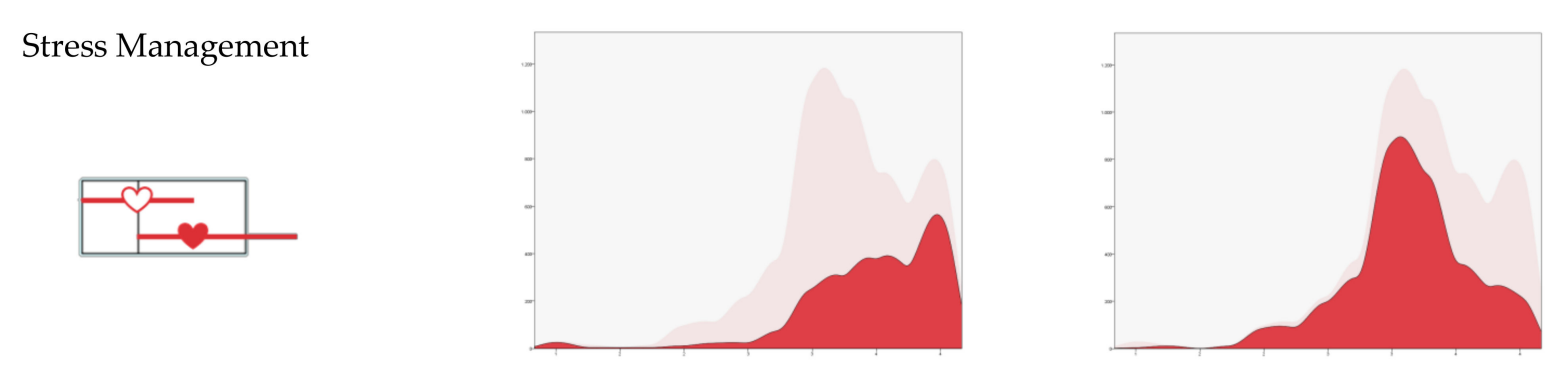
Composition of clusters. Note: Factors in order of importance of input. Cluster comparison in the first column. Note: The *x*-axis corresponds to each of the dimensions of emotional intelligence, and the *y*-axis corresponds to the frequency.

Mean scores of the sample on the emotional intelligence dimensions are shown above in Table 1. The two-stage cluster analysis performed to identify the emotional intelligence profiles found two groups or clusters (Figure 2).

The first cluster (C1), which corresponds to an emotional profile that could be called “high scores on emotional intelligence”, made up of 38.7% of the sample (*n* = 525), was characterized by scores above the overall mean in mood (M = 3.65), adaptability (M = 3.42), intrapersonal (M = 3.23), interpersonal (M = 3.48) and stress management (M = 3.48).

The second cluster (C2), which corresponds to an emotional profile that could be called “low scores in emotional intelligence”, with 61.3% of the sample (*n* = 832), was defined by scores below the sample mean in mood (M = 2.78), adaptability (M = 2.66), intrapersonal (M = 2.37), interpersonal (M = 2.87) and stress management (M = 3.13).

Table 2 shows the descriptive statistics for both clusters and the results of the comparison of means between profiles in perceived mobbing, social support and sensitivity to anxiety (and its physical, cognitive and social components).

Firstly, differences were observed between clusters in perceived mobbing, where C2 had a higher mean score. In line with this, the mean scores of C2 were significantly higher in global anxiety and in its physical, cognitive and social components than in C1.

The differences between clusters in perceived social support revealed significantly higher mean scores in C1 than C2.

### 3.3. Model of Social Support and Anxiety Mediation in the Relationship between Emotional Intelligence and Perceived Mobbing

**Figure 3:**
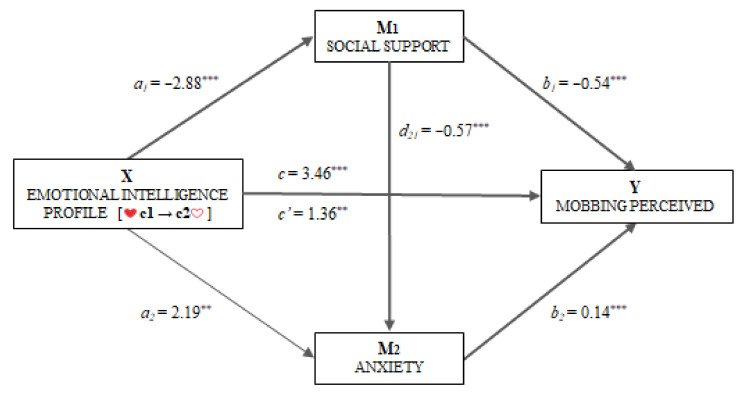
Multiple mediation model of social support and anxiety on the relationship between the emotional intelligence profile and perceived mobbing. ** *p* < 0.01, *** *p* < 0.001.

For computation of the mediation model, belonging to one or the other emotional intelligence profile (coded 0 = C1 and 1 = C2) was taken as the independent variable. Perceived mobbing was proposed as the dependent variable, and, finally, perceived social support (M_1_) and the global anxiety index (M_2_) as mediator variables. Thus, a multiple mediation model with two mediator variables tested their roles and their interaction when operating in series (Figure 3).

First, the existence of a statistically significant effect (*B*_EIp_ = −2.88, *p* < 0.001) of emotional intelligence profile (X) on social support (M_1_) was observed. The second regression analysis took Mediator 2 as the result variable, and the emotional intelligence profile (X) and social support (M_1_) were included in the equation. There was a significant effect of social support (*B*_SS_ = −0.57, *p* < 0.001) and the emotional intelligence profile (*B*_EIp_ = 2.19, *p* < 0.01) on sensitivity to anxiety (M_2_).

In the third regression analysis, the effect of the independent variable and of the two mediators was estimated by taking perceived mobbing (Y) as the result variable. In this case, significant effects were found for the social support (*B*_SS_ = −0.54, *p* < 0.001) and sensitivity to anxiety (*B*_Anx_ = 0.14, *p* < 0.001) mediators, and for the emotional intelligence profile (*B*_EIp_ = 1.36, *p* < 0.01) as the independent variable. The total effect of the emotional intelligence profile on perceived mobbing was significant (*B*_EIp_ = 3.46, *p* < 0.001).

Then, the analysis of indirect effects was carried out using bootstrapping. Even though the effect of emotional intelligence was statistically significant through Paths 2 [Ind_2_: X→M_1_→M_2_→Y; *B* = 0.23, *SE* = 0.06, 95% *CI* (0.12, 0.38)] and 3 [Ind_3_: X→M_2_→Y; *B* = 0.30, *SE* = 0.11, 95% *CI* (0.12, 0.56)], the data suggest that Path 1 [Ind_1_: X→M_1_→Y; *B* = 0.05, *SE* = 0.01, 95% *CI* (0.02, 0.09)] was the most important.

To estimate the importance of each of the paths, contrast or comparison tests of the mediators were applied. The results found that there were statistically significant differences in the contrasts of the indirect effects [Ind_1_ * Ind_2_: 1.32, SE = 0.26, 95% IC (0.82, 1.88)] and [Ind_1_ * Ind_3_: 1.25, SE = 0.29, 95% IC (0.68, 1.86)], while the difference between indirect effects was not statistically significant [Ind_2_ * Ind_3_: −0.07, SE = 0.11, 95% IC (−0.32, 0.14)].

## 4. Discussion

This study found significant associations between the components of emotional intelligence and perceived social support. The higher the score on the interpersonal, mood and adaptability components was, the higher the perceived social support available to nursing personnel, with a large effect size in all the correlations. Data compatible with these were found by Montes-Berges and Augusto [48] and, more recently, by Fernández-Lasarte et al. [49], who demonstrated that emotional intelligence correlated significantly with perceived social support in various samples. However, this did not hold true in the study by Extremera et al. [54] in a sample of teachers, where emotional intelligence did not correlate with perceived social support, while support of coworkers had a mediating effect between emotional intelligence and teacher commitment, improving involvement in their work. More research is therefore necessary to clarify the role of social support and its relationship with emotional intelligence, and the intervention of possible mediator variables.

In the relationship between emotional intelligence and sensitivity to anxiety, the stress management component correlated significantly negatively with the global sensitivity to anxiety score and, in particular, with the cognitive anxiety score, with a large effect size. The findings of other researchers have related higher tolerance and stress management to lower perceived anxiety, where emotional intelligence usually exerts a mediator role between sensitivity to anxiety and manifestation of stress or anxiety symptoms [32,34]. Killgore et al. [34] proposed a model in which sensitivity to anxiety led to less competence in different aspects of the emotional intelligence trait, and not as much emotional skill, specifically with regard to the interpersonal, stress management, adaptability and mood components, which, in turn, increases the probability that a person would respond to physiological sensations with more anxiety.

Another of the objectives of this study was to explore the different emotional intelligence profiles in nursing. The cluster analysis found two profiles based on the emotional intelligence components, which were related to perception of psychological harassment in the workplace and to sensitivity to anxiety.

The first group (38.7% of the sample) comprised nurses with high scores in the intrapersonal, interpersonal, stress management, adaptability and general mood components of emotional intelligence. The second, larger (61.3%) group was made up of nurses with low scores in these emotional intelligence components. The contrast tests between the two groups of nurses and psychological mobbing or sensitivity to anxiety showed that the first group of nurses, with higher scores in emotional intelligence, also scored lower in perception of psychological harassment and perceived fear/anxiety than the second group. High scores in emotional intelligence were associated with low scores in mobbing and sensitivity to anxiety. In this vein, some authors have suggested that improvement in emotional competence could reduce mobbing [35], perhaps because it has been empirically demonstrated that emotional intelligence exerts a mediating role in coping with stress and anxiety [34]. Other authors have shown that sensitivity to anxiety impedes emotional competence, and the person is more exposed to more anxious responses to physiological sensations [32].

This study also met its objective of developing a mediation model for mobbing in nursing, considering the existence of a significant relationship between emotional intelligence profiles and psychological harassment in the workplace, which included the role of the sensitivity to anxiety and perceived social support psychological variables. Our results showed that perceived social support and sensitivity to anxiety were mediators in this relationship between the variables in the healthcare context, although perceived social support acquired more importance. Thus, perceived social support acted as a buffer in the relationship between emotional intelligence and mobbing. Several of the studies reviewed also found a role of social support in mobbing [46,47] in sustaining affect and improving nurses’ job satisfaction by buffering the adverse effects of workplace violence and mobbing by coworkers [63].

This study went one step further, showing the mediator role of social support in a concrete model and demonstrating its relationship with mobbing. Social support is, then, once more, very important in the relationship established between nurses’ emotional intelligence and mobbing, mediating and strengthening its effect.

### 4.1. Limitations

Some limitations should be kept in mind. Firstly, the sample was made up of a majority of women, and, although this is a reflection of the professional profile, the results may not be generalizable to both sexes. Self-report measures were used in the evaluation of both social support and mobbing [40,55,58], which could increase the correlations between variables. It would be advisable to complete these measures with other instruments based on observation and with contributions from other significant persons in the participants’ setting. As the study design was cross-sectional, no causal relationships could be established between the variables (emotional intelligence, sensitivity to anxiety, perceived social support and mobbing) analyzed; however, the trend in their relationships may be inferred.

### 4.2. Future Investigation

A future line of research will be to study how much is explained by personal variables, such as the role of positive and negative affect, tolerance to frustration and stress coping mechanisms, as well as continuing with the study of preventive strategies and characteristics that strengthen the organization to avoid circumstances associated with mobbing and its high psychological and economic costs. Finally, not forgetting the organizational side, we propose meeting spaces, which would favor work relations, training programs for acquiring emotional intelligence competencies, among many other activities in the organization to promote social support and staff relations. It would also be of interest to check whether the mediation model proposed can be applied to other groups where there is a high prevalence of mobbing, such as among workers in large companies.

### 4.3. Practical Implications

The results of this study have implications for professional practice, leading to both prevention of mobbing in the workplace and intervention when it occurs. Our results advise reinforcing the social support network of nursing personnel to improve the work climate, with the organizational and personal measures that this involves, and reinforcing the emotional intelligence of nurses through university and on-the-job programs [15]. We agree with Keskin et al. [11] that those who have a greater ability to perceive, understand, use and manage their own and others’ emotions are more likely to be psychologically well-adjusted. We also agree with Wan et al. [30] that emotional intelligence facilitates coping with the challenges of the job and achieving goals, although neurological, genetic and contextual factors also intervene in self-regulation and emotional control. Relaxation or mindfulness strategies may reduce sensitivity to anxiety and help cope with stress, as suggested by other authors (e.g., [30]). Lastly, it is worth mentioning how important it is for business leaders to make this problem, which silently affects their workers, known and improve the perception of support of mobbing victims.

## 5. Conclusions

These findings have important implications for understanding psychological harassment in the workplace. Keeping in mind the severe psychosocial consequences of mobbing at work, these data underline the urgent need to contribute to the positive development of the organization and its members by improving interpersonal relations and providing a place for staff to interact.

High sensitivity to anxiety and low emotional intelligence are associated with a greater presence of mobbing at work. Mobbing can be buffered if the person perceives sufficient support from family, friends or significant others. Therefore, contributing to social support as a protective factor and reducing sensitivity to anxiety as a possible risk factor are the two main lines of intervention derived from this study. We also suggest continuing with the study of the role of positive affect and satisfaction in the workplace as possible mediators in the relationship between emotional intelligence and mobbing.

## Figures and Tables

**Table 1:** Perceived mobbing, emotional intelligence, social support and sensitivity to anxiety. Bivariate correlations (*n* = 1357).

	1	2	3	4	5	6	7	8	9	10	11
1. Perceived Mobbing	-										
2. Intrapersonal	−0.12 ***	-									
3. Interpersonal	−0.11 ***	0.47 ***	-								
4. Stress Management	−0.21 ***	0.06 *	0.11 ***	-							
5. Adaptability	−0.18 ***	0.58 ***	0.70 ***	0.15 ***	-						
6. Mood	−0.23 ***	0.51 ***	0.54 ***	0.21 ***	0.66 ***	-					
7. Social Support	−0.30 ***	0.31 ***	0.38 ***	0.17 ***	0.38 ***	0.50 ***	-				
8. Global Anxiety	0.24 ***	−0.06 *	−0.04	−0.34 ***	−0.10 ***	−0.18 ***	−0.22 ***	-			
9. Physical anxiety	0.20 ***	−0.03	−0.06 *	−0.29 ***	−0.10 ***	−0.16 ***	−0.21 ***	0.92 ***	-		
10. Cognitive anxiety	0.21 ***	−0.04	−0.08 **	−0.35 ***	−0.12 ***	−0.20 ***	−0.26 ***	0.92 ***	0.83 ***	-	
11. Social anxiety	0.24 ***	−0.09 ***	0.02	−0.27 ***	−0.06 *	−0.14 ***	−0.15 ***	0.87 ***	0.67 ***	0.68 ***	-
*M*	26.45	2.70	3.11	3.26	2.96	3.11	30.05	12.97	3.69	2.81	6.47
*SD*	9.24	0.70	0.52	0.53	0.56	0.64	4.05	11.80	4.53	4.04	4.45

* *p* < 0.05; ** *p* < 0.01; *** *p* < 0.001. Note. 1: Perceived Psychological Harassment Questionnaire (CAPP); 2–6: Brief Emotional Intelligence Inventory (EQ-i-20M); 7: Brief Perceived Social Support Questionnaire (CASPE); 8–11: Anxiety Sensitivity Index-3 (ASI-3).

**Table 2:** Perceived mobbing, social support and anxiety. Descriptive statistics and t-test by emotional intelligence profile.

	Emotional Intelligence						t	d
	c1			c2				
	N	Mean	SD	N	Mean	SD		
Perceived mobbing	525	24.34	9.04	832	27.34	9.13	−6.80 ***	0.38
Social support	525	31.81	2.83	832	28.92	4.31	14.82 ***	0.83
Global Anxiety	525	10.62	11.67	832	14.46	11.65	−5.90 ***	0.33
Physical anxiety	525	2.88	4.46	832	4.21	4.50	−5.32 ***	0.30
Cognitive anxiety	525	2.01	3.87	832	3.32	4.06	−5.97 ***	0.33
Social anxiety	525	5.74	4.46	832	6.93	4.39	−4.85 ***	0.27

*** *p* < 0.001.

## Data Availability

The data that support the findings of this study are available from the corresponding author upon reasonable request.
